# Prospective randomized controlled trial on the safety and neuroprotective efficacy of remote administration of hypothermia over spleen during acute ischemic stroke with mechanical thrombectomy: rationale, design, and protocol

**DOI:** 10.3389/fneur.2024.1382365

**Published:** 2024-07-16

**Authors:** Honglian Duan, Zhe Cheng, Xiaokun Geng, Gary B. Rajah, Jie Gao, Yang Guo, Lipeng Cai, Yanna Tong, Fengwu Li, Qian Jiang, Zhenzhen Han, Yuchuan Ding

**Affiliations:** ^1^Department of Neurology and Stroke Center, Beijing Luhe Hospital, Capital Medical University, Beijing, China; ^2^Luhe Institute of Neuroscience, Capital Medical University, Beijing, China; ^3^Department of Neurosurgery, Wayne State University School of Medicine, Detroit, MI, United States; ^4^Department of Neurosurgery, Munson Medical Center, Traverse City, MI, United States

**Keywords:** spleen immune reaction, brain inflammation, large vessel occlusion, ineffective recanalization, neuroprotection

## Abstract

**Background:**

Brain inflammation plays a key role in ischemia/reperfusion (I/R) injury and is the main cause of “ineffective or futile recanalization” after successful mechanical thrombectomy (MT) in acute ischemic stroke (AIS). One of the primary sources of inflammatory cells after AIS are derived from the spleen. As an innovative and potential neuroprotective strategy after stroke, Remote Administration of Hypothermia (RAH) temporarily suppresses immune activities in the spleen, reduces the release of inflammatory cells and cytokines into blood, and thus reversibly diminishes inflammatory injury in the brain.

**Methods:**

This single-center, prospective, randomized controlled study (RCT) is proposed for AIS patients with anterior circulation large vessel occlusion (LVO). Subjects will be randomly assigned to either the control or intervention groups in a 1:1 ratio (*n* = 40). Participants allocated to the intervention group will receive RAH on the abdomen above the spleen prior to recanalization until 6 h after thrombectomy. All enrolled patients will receive standard stroke Guideline care. The main adverse events associated with RAH are focal cold intolerance and abdominal pain. The primary outcome will assess safety as it pertains to RAH application. The secondary outcomes include the efficacy of RAH on spleen, determined by spleen volumes, blood inflammatory factor (cells and cytokines), and on brain injury, determined by infarction volumes and poststroke functional outcomes.

**Discussion:**

This study aims to examine the safety and preliminary effectiveness of RAH over the spleen during endovascular therapy in AIS patients. The results of this study are expected to facilitate larger randomized clinical trials and hopefully prove RAH administration confers adjuvant neuroprotective properties in AIS treated with MT.

**Clinical trial registration:**

https://www.chictr.org.cn/. Identifier ChiCTR 2300077052.

## 1 Introduction

Mechanical thrombectomy (MT) is recommended for acute ischemic stroke (AIS) patients with large vessel occlusion (LVO) due to high revascularization rates. However, almost half of the stroke patients who undergo successful revascularization still experience severe disability or death. This phenomenon has been known as “ineffective or futile recanalization” (1–3). It is crucial to develop neuroprotection strategies to protect the brain in addition to endovascular therapy.

An increasing number of studies have demonstrated that splenic-derived inflammatory cells are an important source of inflammatory infiltration within brain tissue and that post-stroke activation of splenic inflammatory cells invade brain tissue, exacerbating the inflammatory and thus ischemia/reperfusion(I/R) injury (4–7). It was reported that splenic contraction occurs in some stroke patients within 24–48 h, followed by gradual recovery of splenic volume with clinical function improvement, according to clinical studies (8). Animal experiments further confirmed a significant reduction in spleen volume during the acute phase of stroke and the transit of splenocytes from the spleen to the ischemic area in the brain (8, 9). These studies suggested that activation and release of splenic inflammatory cells were the key factor in exacerbating cerebral ischemia/reperfusion injury. Inhibition of the reaction may block the inflammatory cascade response in the brain, leading to neuroprotection. Because of the detrimental effects of splenic inflammatory cells and factors, splenectomy is a widely studied means of splenic suppression. Splenectomy in a mouse model resulted in lower blood levels of multiple inflammatory factors than normal mice (9), reducing inflammatory cell infiltration in the brain and reducing brain infarct volume (10). Splenic pulse treatment suppressed the immune response in septic rats (11), and splenic irradiation similarly reduced infarct volume in mice (12). However, these approaches are largely prohibited in clinical practice since they cause irreversible suppression of the spleen (10, 13, 14). In the current study, the RAH above the spleen is hypothesized to prevent splenic contraction and reduce the release of splenic inflammatory cells and cytokines, which in turn may decrease brain infarct volume and improve long-term neurological function after stroke in humans. Furthermore, before this proposed clinical trial, our team investigated the efficacy of Remote Administration of Hypothermia (RAH) to the spleen in animal models of middle cerebral artery occlusion. During the experiment, the spleen temperature was reduced to 27°C, leading to reduced brain inflammatory response, decreased infarct volume, and improved functional prognosis (unpublished data). While our original pre-clinical animal data remains unpublished, the connection between splenic response and stroke injury, as well as protective effects, has been discussed in a recent study. In this study, it was demonstrated that diminished splenic response through inactivation or knockout of GPR55 improved neurological outcomes in a rodent model (15).

## 2 Methods and analysis

### 2.1 Study design

This is a single-center prospective randomized controlled trial of AIS patients with LVO in the anterior circulation. We will determine the safety and preliminary efficacy of RAH in AIS patients with LVO in the anterior circulation with concurrent recanalization by MT. All participants or proxies will be informed of the potential risks and possible benefits and consent to participate in this study. This study was approved by the regional ethics committee and registered at www.chictr.org.cn (ChiCTR 2300077052).

Participants assigned to the intervention group will receive RAH prior to recanalization until 6 h after thrombectomy. All participants from either group will be given concurrent standard-of-care therapies consistent with the Guidelines for Stroke Management.

### 2.2 Participants and screening

**Figure 1:**
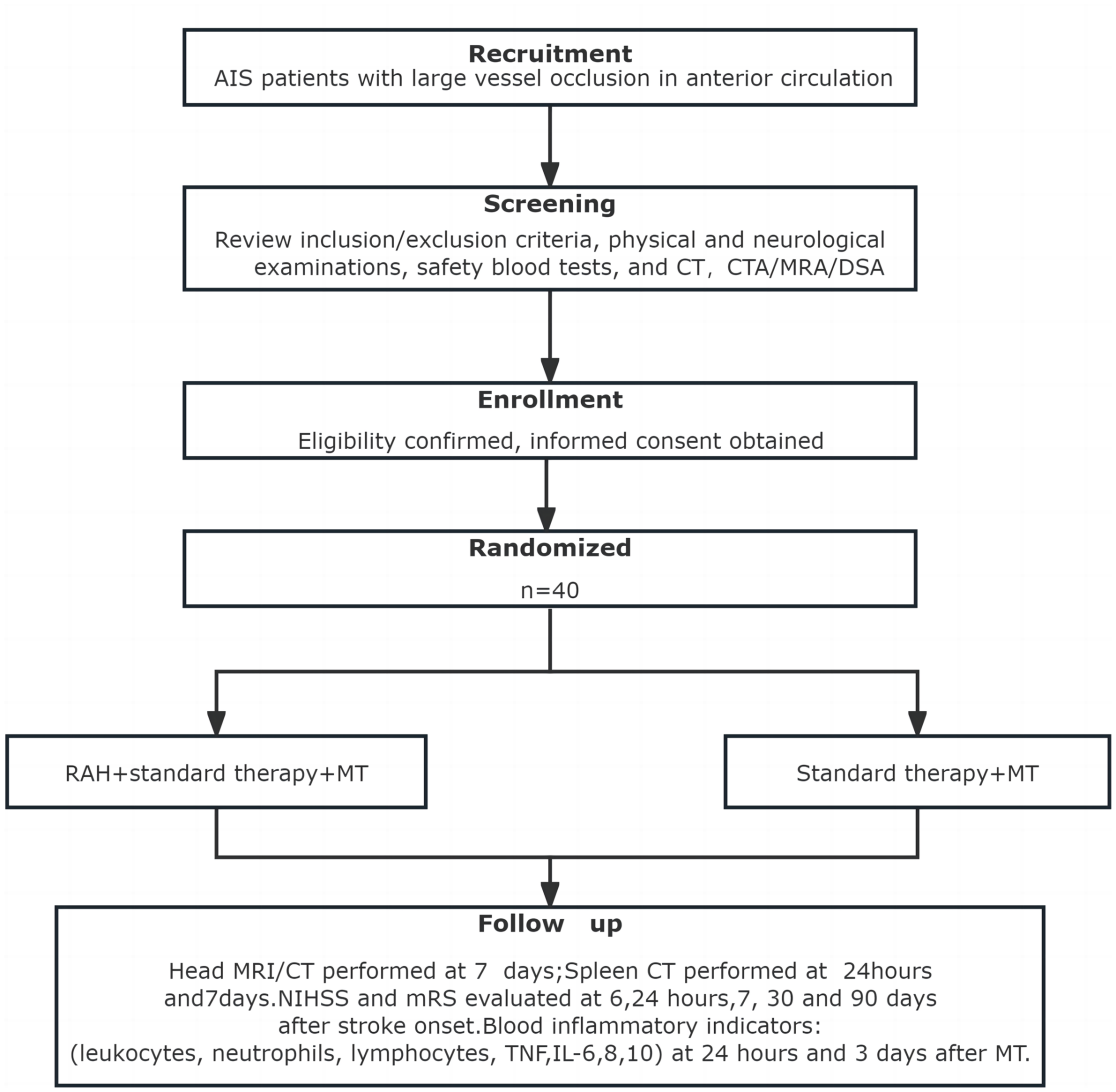
Flowchart of the study.

Participants will be recruited from the Stroke Intervention & Translational Center (SITC) in Beijing Luhe Hospital, Capital Medical University. Eligible patients will be randomly assigned to RAH or control group when they fulfill the inclusion criteria but not the exclusion criteria. The flowchart of the study is in Figure 1.

The criteria for recruitment are as follows: (1) Age of 18–80 years; (2) Patients with AIS; (3) LVO of the anterior circulation confirmed by computer tomography angiography (CTA), magnetic resonance angiography (MRI), or digital subtraction angiography (DSA); (4) Baseline National Institute of Health stroke scale (NIHSS) score ≥ 6 and Alberta Stroke Program Early CT Score (ASPECTS) >5; (5) Less than 24 h from stroke onset; (6) Acute occlusion of intracranial or extracranial segments of the anterior circulation vessels (thrombolysis in cerebral infarction score [TICI] classification 0-I); and (7) Informed consent of the participant or legally authorized representative (in cases where the patient’s decision-making ability is impaired due to a stroke) will be obtained.

The criteria for exclusion are as follows: (1) Spontaneous revascularization or rapid spontaneous improvement in neurological function (NIHSS<6 points); (2) Unsuccessful revascularization with MT (TICI = 0); (3) Rapid improvement in symptoms after revascularization; (4) Cranial imaging showing infarct area exceeding one-third of the area supplied by the middle cerebral artery pre-MT; (5) Pre-stroke modified Rankin Scale (mRS) >2; (6) Blood glucose <2.7 or > 22.2 mmol/L; (7) Laboratory evidence of coagulation dysfunction (platelet count <40 × 10^9^/L, APTT >50 s, or INR >3.0); (8) Pregnancy; (9) Splenic-related hematologic disorders, hypersplenism, or suppression; (10) Hypersensitivity of the skin, such as cold urticaria; (11) Obesity of patients (BMI ≥28/waist circumference > 90 cm); (12) Abdominal injury, surgical trauma and other factors intolerance to splenic hypothermia; (13) Patients enrolled within 3 months prior to this clinical trial or already enrolled in another clinical trial; (14) Failure to obtain informed consent; and (15) Patients with Splenectomy.

### 2.3 Randomization and blindness

Participants will provide consent and then be randomly assigned to one of the two groups in a 1:1 (*n* = 40) fashion using computer-generated randomization. Patients will be randomized into the groups using opaque randomization envelopes. The nurse who is not blinded will perform RAH and temperature monitoring. After the case observation, neurological function scoring, data collection, and statistical analysis will be performed by the researchers who are blinded to the treatments.

### 2.4 Interventions

**Figure 2:**
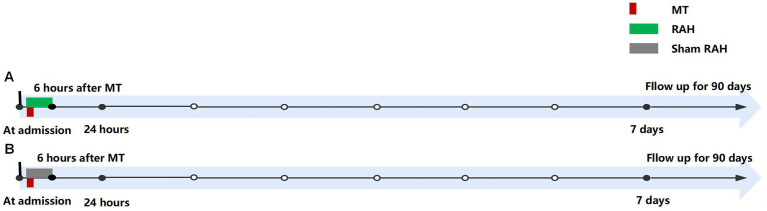
Procedure timeline for protocol. **(A)** Intervention group; **(B)** Control group. MT: mechanical thrombectomy; RAH, remote administration of hypothermia.

**Figure 3:**
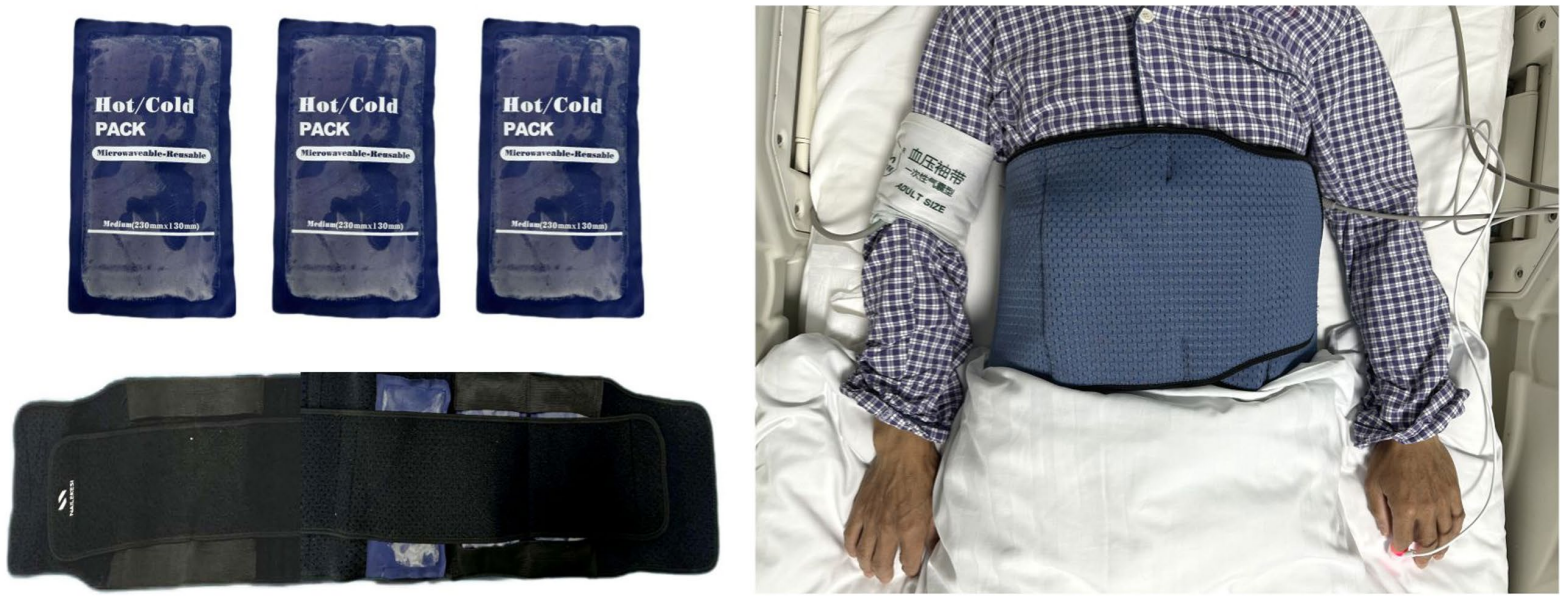
Administration of hypothermia (RAH) on the spleen.

Both groups will receive standard treatments according to the AIS guidelines. Within the intervention group, patients will initiate RAH prior to recanalization until 6 h after thrombectomy (Figure 2). RAH is achieved by applying ice packs to the spleen area (left side of the abdomen and left back) and secured with an abdominal belt (Figure 3). A temperature probe will be used to continuously monitor the skin temperature of the left abdomen, indirectly inferring the spleen temperature. The ice packs will be changed every hour to keep the skin temperature at hypothermic level (25°C). In the study, soft towels will be placed between ice pack and skin and the thickness of towels will be adjusted to maintain the temperature at approximately 25°C. The thickness of towels has been previously explored in a pre-experiment in healthy human individuals (eight healthy individuals applying RAH for 6 hours). Based on prelim towel placement between ice packs: patients should not have abdominal pain, diarrhea, local frostbite, rash, and chills. The axillary temperature will be monitored hourly. Vital signs (e.g., blood pressure, heart rate, body temperature, respiratory rate) of patients will be monitored every 15 min. Meanwhile, any abdominal symptoms or discomfort will be closely monitored. The RAH will be stopped immediately if the patient develops a tendency for adverse reactions and complications.

Patients in the control group (Sham RAH) will apply packs at room temperature in the left abdomen until 6 h after thrombectomy (Figure 2).

### 2.5 Outcomes and assessment procedures

#### 2.5.1 Primary outcome (safety)

The primary outcome is safety, defined as any adverse events related to RAH. The main adverse events associated with RAH are focal cold intolerance and abdominal pain. Other adverse events include abdominal skin damage and/or discomfort, such as diarrhea, local frostbite, rash, and chills. All adverse events will be determined independently by trained medical team personnel who are blinded to the randomized groups.

#### 2.5.2 Secondary outcomes (efficacy)

(1) Effect of RAH on Spleen: (a) spleen volume measurement: Spleen CT will be performed preoperatively, 24 h and 7 days after MT to determine the change in spleen volume. The area of each layer of the spleen will be calculated by circling the area with the Siemens CT workstation image software, and the overall spleen volume will be calculated by superimposing the layer thicknesses. An ultrasound of the spleen will be performed alternatively if the CT is not done. (b) comparison of blood inflammatory profiles, the changes in inflammatory cells and inflammatory factors (including leukocytes, neutrophils, lymphocytes, tumor necrosis factor, and interleukins 6, 8, and 10, etc.) before, at 24 h and 7 days after MT will be completed. (2) Effect of RAH on Brain: (a) infarct volume (MRI-DWI) between the two groups at 7 days after MT. Infarct volume measurement of cerebral infarcts will be performed by MRI-DWI with a Siemens Syngo workstation. The area of each infarcted layer will be automatically derived by circling the region of interest (ROI) on software of the workstation, and then the overall infarct volume is calculated by superimposing the layer thickness; (b) Intracranial hemorrhage will be classified as symptomatic intracranial hemorrhage based on the following: lethal intracranial hemorrhage and symptomatic intracranial hemorrhage defined as a postoperative CT showing an intracranial hemorrhagic lesion consistent with an NIHSS deterioration score of ≥4 according to the European-Australasian Acute Stroke Study (ECASS II) criteria; (c) comparison of neurological functional independence evaluation at 90-days for functional independence ratio (mRS 0–2 score) and 90-day mortality (mRS 6 score) between RAH and control groups will be determined.

### 2.6 Estimation of sample size

This is a pilot study of the safety of RAH with MT in AIS patients with LVO of anterior circulation. There are no similar clinical studies like this, and thus, no information is available. According to Hertzog (16), 10–20 patients in each group were sufficient to assess the feasibility of a pilot study. Dobkin suggested that a minimum of 15 patients per group would be sufficient to decide whether a larger multicenter trial should be conducted (17). Therefore, we aimed to recruit 20 patients for each group in this study.

### 2.7 Data management, monitoring, and quality assessment

The investigator will enter data accurately and promptly into the case report form based on the original observation records of subjects. Data will be entered using a dual-machine system and be verified twice for accuracy We will apply the multiple imputation method to process missing values. However, our primary focus during the implementation and data management stages of the clinical trials will be to prevent the occurrence of missing data as much as possible. We will strengthen data collection efforts and avoid any missing data cases to ensure our dataset’s integrity and effectiveness. Issues will be promptly reported to the monitor. Case report forms will be filed, stored in numbered order and archived.

The Luhe Hospital Research Expert Steering Committee and Ethics Committee will provide follow-up and supervision for the research project. The Luhe Hospital Research Expert Steering Committee will conduct quality control of the study, including planned enrollment, screened cases, currently enrolled cases, enrollment rate calculation, and follow-up management. The committee will analyze any problems and their causes and implement appropriate measures to manage the study. This study will follow standardized procedures for implementation to ensure the scientific rigor of the research and the accuracy, completeness, and authenticity of the data. These will include managing all aspects of the study, such as the informed consent process, screening and enrollment, randomization management, blinding, protocol adherence, management and recording of research instruments, data collection, project implementation, and management of project documents. The third-party quality control team will conduct regular on-site monitoring visits to hospitals to ensure strict adherence to all elements of the study protocol and accurate completion of study data. The project will be monitored and audited systematically, with a Quality Control report provided to the project leader. Additionally, 20% of the raw data will be spot-checked to examine data authenticity and completeness.

### 2.8 Safety measurement

Due to the small number of participants, the short follow-up period, and the RAH applied to the skin surface of the splenic region, this study is considered relatively safe with few adverse reactions. Therefore, no interim analysis of safety will be performed. Special adverse events and serious adverse events will be closely monitored throughout the study. Any unfavorable medical event that occurs from the beginning of patient enrollment to follow-up, regardless of whether it is causally related to the trial treatment, will be judged as an adverse event. The investigators will evaluate these potential events according to their causality and severity. If they occur, they will be reported to the Ethics Committee of the Luhe Hospital and make amends.

### 2.9 Statistical analyses

Data will be entered by persons not involved in the test, using double entry and setting up data range checks. Statistical analysis will be performed using SPSS version 19 (SPSS Inc., Chicago, IL, United States). Categorical variables will be expressed as percentages, and differences will be compared using the chi-square test; continuous variables conforming to normal distribution will be expressed as mean ± standard deviation, and differences will be compared using a *t*-test or ANOVA; continuous variables not all conforming to normal distribution will be expressed as median and upper and lower quartiles, and differences will be compared using rank sum test. Multi-factor linear regression or multi-factor Logistic regression analyses will be performed to control confounding factors according to the data type and outcome. *p* < 0.05 will be considered statistically significant.

### 2.10 Patient and public involvement

Patients were not involved in the development of the study protocol and design, which were reviewed, revised and approved by the Research Expert Steering Committee of the Luhe Hospital and the Ethics Committee of the Luhe Hospital. The results of the study will be disseminated to the general public by means of seminars, public presentations and publication in peer-reviewed journals.

## 3 Discussion

Revascularization strategies in acute ischemic stroke are becoming increasingly sophisticated, while successful revascularization gives ideal functional prognosis in only 46% of patients (1). Inflammation during reperfusion plays a significant role in I/R injury, hindering effective recanalization (18–20). Studies have identified splenic inflammatory cell activation and release after stroke as an important source of the central immune response (4, 18, 21). One recent study demonstrated that neurons in the central amygdala and paraventricular nucleus of the brain act directly over the spleen to regulate plasma cell production and that splenic denervation inhibits this process (22). The results suggested a regulatory role of brain-spleen circulation on adaptive immunity. Many preclinical studies proposed that activation and release of splenic inflammatory cells are closely related to cerebral I/R injury. Inhibition of these processes reduced brain inflammation, and thus attenuated the I/R injury (4, 5, 7, 9, 23, 24).

### 3.1 Spleen-derived immune response and neuroprotective effects of inhibiting splenic inflammation

After a stroke, the brain and spleen communicate through various pathways, triggering immune cells to release cytokines and migrate to the affected area of the brain (25). These cytokines increase in the bloodstream around 24 h after the onset of ischemia. Levels of TNF-α, TNF-γ, IL-6, MCP-1, and IL-2 were significantly increased at 6 and 22 h after MCAO in mice with stroke injuries (26, 27). Besides, chemokines CCL2 and CCL3 attract monocytes and neutrophils after a stroke, with CCL2 peaking 2 days (28–30). Suppressing the splenic immune response after a stroke may be neuroprotective. Diminished splenic response by inactivation or knockout of GPR55 has been shown in rat models to improve neurological outcomes (15). Gu et. Al reveals external rat cooling to 30C with ETOH topically can result in neuroprotection and inhibit splenic contraction (31). Vagal nerve stimulation of the spleen has also been shown to reduce splenic inflammatory response (32).

Previous studies demonstrated that splenectomy prior to or immediately after a stroke significantly reduces infarct volume and inflammatory cell infiltration post-reperfusion in the MCAO model (10). Additionally, splenic pulsed therapy was found to suppress the immune response (11), non-surgical irradiation of the spleen in rats after MCAO reduced infarct volume (12) and inhibition a subpopulation of spleen-derived pro-inflammatory macrophages in mice resulted in a significant reduction in brain infarct volume (33).

### 3.2 Restrictions of permanent splenic suppression and traditional hypothermia

Splenectomy, spleen pulse ultrasound, or splenic radiation reduced brain inflammation in the ischemic region, while this immunosuppressive process is irreversible. These methods that permanently inhibit splenic inflammation have harmful effects on immune cell proportion imbalance, and bacterial infection risk increase. These side effects outweigh their neuroprotective effects (7, 10, 13), limiting their clinical application. Methods of transient suppression of splenic inflammation during the peak of ischemia–reperfusion may become a promising therapeutic tool.

Hypothermia has long been considered a promising approach to neuroprotection. Nevertheless, the application of post-stroke hypothermia is extremely restricted and difficult to translate into clinical practice (34). The common means of hypothermia after craniocerebral injury include local hypothermia and systemic hypothermia. Local hypothermia in the brain is less effective and the high incidence of adverse events with systemic hypothermia (35, 36) has greatly limited its clinical dissemination. Endovascular hypothermia based on revascularization with an invasive nature has shown a favorable trend in neuroprotection, while the high cost and demanding invasive operational procedure restrict its clinical application (37).

### 3.3 Improved neurological function by reducing splenic immune reaction with hypothermia

Animal experiments found that systemic mild hypothermia can reduce the splenic immune response (31). Moderate hypothermic exposure (34°C, 1.5 h) of splenocytes co-cultured with rat primary neurons (38) shows a protective effect of hypothermia-cultured splenocytes on oxyglucose-deprived neurons. Before this proposed clinical trial, our team has determined the efficacy of Remote RAH on middle cerebral artery occlusion animal models. RAH was induced by cold pad that was placed above the rat abdomen. During the experiment, the spleen temperature was monitored by a temperature probe directly, and the temperature was reduced to 27°C. The experiment demonstrated that RAH could inhibit the reduction of splenic volume, reduce brain inflammatory response, decrease infarct volume, and improve functional prognosis (submitted, unpublished data). In addition, the splenic weight in MCAO was increased by RAH, suggesting that splenic hypothermia may inhibit splenic contraction and weight loss by reducing inflammatory cell release. It must be acknowledged that the optimal temperature for RAH remains to be determined. In our animal experiment, the lowest temperature of the spleen was 27°C. It is challenging to ascertain the temperature of a human spleen without direct invasive means. Our previous temperature explorations found that the average skin temperature in the splenic region was around 25°C and did not cause significant discomfort. The results of this trial will help determine the most optimal temperature.

### 3.4 Adjuvant neuroprotective strategy with RAH in addition to recanalization

As research progresses, the integration of neuroprotective measures with thrombolysis or thrombectomy is increasingly perceived as a pivotal step toward evolving comprehensive neuroprotective strategies for AIS (39, 40). Recent strides in MT for AIS have unveiled opportunities to effectively utilize additional neuroprotective treatments post-reperfusion (24, 41). Notable approaches include NA1 [an inhibitor of the excitotoxic post-synaptic density protein 95 (PSD-95)] (42), intra-arterial cooling infusion (37), and high-flow normobaric oxygen (NBO) (43). Drawing information from the neuroprotective influence of RAH over the spleen as observed in ischemic stroke animal models (submitted, unpublished data), our team would like to pioneer a clinically practical RAH methodology. This technique is geared toward establishing RAH’s neuroprotective effectiveness in AIS patients following endovascular recanalization. Our approach synergistically combines reperfusion and neuroprotection strategies, including recanalization therapy, hypothermia, and splenic inflammation inhibition. The goal is to mitigate I/R injuries and strengthen neurological function. We postulate that the combined regimen of RAH and MT might improve endovascular treatment outcomes more than MT alone. Such a combination could potentially enhance the functional outlook for AIS patients and diminish complications, including bleeding, disability, and mortality, especially in severe acute cerebral infarction cases. This comprehensive approach provides an optimized treatment model while also promoting healthcare efficiency and cost savings. By potentially reducing unnecessary medical costs, especially when using cost-effective methods like ice packs, it aligns with the broader goal of economical healthcare. Our method of regional cooling is not only user-friendly and effective but also versatile, hinting at its potential use in hypothermic treatments for different diseases and targeted organ therapies.

### 3.5 Limitations

First, this pilot study will be conducted at a single center with a small sample size, which may limit the generalizability of the findings. Secondly, it is very difficult, if not impossible, to cool the spleen exclusively or to monitor human spleen temperature directly, and the optimal duration and target temperature of RAH remains to be determined. Thirdly, the effect of RAH over the spleen will be inferred indirectly by measuring spleen volume with CT and inflammatory factors in the blood, rather than through direct observation. Lastly, the lack of blinding for the nurse administering the RAH could introduce bias, although this is unlikely to influence the data analysis and final results.

## 4 Conclusion

This proposal attempts to confirm the safety and preliminary efficacy of RAH over the spleen and brain after stroke. The results are expected to demonstrate that RAH is safe for clinical use and has a neuroprotective effect by reversibly inhibiting splenic inflammation. This will provide a novel neuroprotective strategy. Preliminary findings will be used to set parameters for future clinical studies.

## Ethics statement

The present study has received approval from the ethics committee of Luhe Hospital, Capital Medical University, Beijing, China (2023-LHKY-068-02). Prior to their inclusion in the study, all participants are required to provide informed consent.

## Author contributions

HD: Writing – original draft. ZC: Writing – original draft. XG: Conceptualization, Funding acquisition, Writing – review & editing. GR: Writing – review & editing. JG: Writing – review & editing, Data curation. YG: Writing – review & editing, Supervision. LC: Writing – review & editing, Methodology. YT: Writing – review & editing, Formal analysis. FL: Writing – review & editing, Investigation. QJ: Writing – review & editing, Methodology. ZH: Writing – review & editing, Project administration. YD: Writing – review & editing.
